# Alarming increase in tuberculosis and hepatitis C virus (HCV) among HIV infected intravenous drug users

**DOI:** 10.7448/IAS.17.4.19625

**Published:** 2014-11-02

**Authors:** Cristiana Oprea, Irina Ianache, Roxana Radoi, Simona Erscoiu, Gratiela Tardei, Olimpia Nicolaescu, Maria Nica, Petre Calistru, Simona Ruta, Emanoil Ceausu

**Affiliations:** 1 HIV, Victor Babes Clinical Hospital for Infectious and Tropical Diseases, Bucharest, Romania; 2 Laboratory of Immunology and Virology, Victor Babes Clinical Hospital for Infectious and Tropical Diseases, Bucharest, Romania; 3 Pneumology and Tuberculosis, Victor Babes Clinical Hospital for Infectious and Tropical Diseases, Bucharest, Romania; 4 Laboratory of Bacteriology, Victor Babes Clinical Hospital for Infectious and Tropical Diseases, Bucharest, Romania; 5 Infectious Diseases, Victor Babes Clinical Hospital for Infectious and Tropical Diseases, Bucharest, Romania; 6 Virology, Stefan S Nicolau Institute of Virology, Bucharest, Romania

## Abstract

**Introduction:**

In the last years, we observed an alarming increase in the number of newly diagnosed HIV infected intravenous drug users (IDUs) co-infected with hepatitis viruses or with severe bacterial infections. The aim of our study was to assess the incidence, the demographic and clinical characteristics of IDUs diagnosed with HIV, HCV and tuberculosis (TB).

**Materials and Methods:**

Prospective study on HIV infected IDUs with HCV and TB admitted in a single centre between January 2009 and April 2014. Data were compared to a group of HIV infected IDUs without TB. Statistical analysis was performed using Graphpad Prism 4.01.

**Results:**

Out of 450 HIV infected IDUs, 134 (29.7%) were diagnosed with HIV, HCV and TB. TB incidence among IDUs increases from 0% in 2009 to 30.2% in 2013. The TB coinfected patients had a mean age at diagnosis of 30 [15–56] years; were in majority males, 106 (84.4%); from urban areas, 120 (89.5%); and had significantly lower education level (85% vs 68.3%, p<0.0001) and higher rates of unemployment (80% vs 55%, p<0.0001) than those without TB. The median CD4 cell count was lower in the TB versus non TB IDUs (143 vs 472/mm^3^, p<0.0001). TB infected IDUs tend to be more frequently late presenters (59.7 vs 24.6, p<0.0001) and to have advanced HIV disease (47.7 vs 7.59%, p<0.0001) than those without TB. TB cultures were positive in 64 (47.7%) patients, 3 (2.2%) had multidrug resistant TB and 2 (1.5%) had extended drug resistance. Disseminated and/or extrapulmonary TB was diagnosed in 51 patients (38%). The overall mortality rate was higher in TB compared to non TB IDUs (19.4% vs 8.2%, p=0.0007), disseminated TB being associated with the most severe immunosuppression (median CD4 cell count 42/mm^3^) and the highest mortality rate (27.4%).

**Conclusions:**

The incidence of TB in HIV/HCV coinfected IDUs was high and rose over the time. TB infection was more frequent in patients with severe immunosuppression and the mortality rate was higher in IDUs with disseminated and/or extrapulmonary disease. IDUs are important candidates for acquiring and transmitting HIV infection, viral hepatitis and TB, being difficult to control due to their high-risk behaviours. Strengthening of HIV transmission prevention strategies, particularly in identified risk groups, is mandatory.

